# Verruköser exophytischer Tumor der Glans penis

**DOI:** 10.1007/s00105-022-05085-3

**Published:** 2022-12-13

**Authors:** Valentin Aebischer, Stephan Forchhammer

**Affiliations:** Universitäts-Hautklinik Tübingen, Eberhard-Karls-Universität, Liebermeisterstr. 25, 72076 Tübingen, Deutschland

**Keywords:** Verruciformes Xanthom, Wart on fire, CHILD-Syndrom, Peniskarzinom, Histologie, Verruciform xanthoma, Wart on fire, CHILD syndrome, Penile cancer, Histology

## Abstract

Ein 59-jähriger Patient stellte sich mit einem seit 1 Jahr wachsenden Tumor an der Glans penis vor, den wir exzidierten. Histologisch zeigte sich eine akanthotische Epidermis, unter der die Papillarkörper prall angefüllt waren mit zahlreichen schaumigen Histiozyten. Der Befund entspricht einem verruziformen Xanthom. Die Differenzialdiagnose eines Peniskarzinoms erfordert eine deutlich radikalere Therapie. Da mutilierende Penisoperationen mit erheblichen psychosexuellen Belastungen für die Patienten einhergehen, kann die Kenntnis dieser gutartigen Diagnose einem vorschnell aggressiven Vorgehen bei ähnlichen Tumoren vorbeugen und zu bedachtem Vorgehen anregen.

## Abstract (en)

A 59-year-old man presented with a growing tumor on the glans penis, which we excised. Histologically, there was an acanthotic epidermis under which the papillary dermis was filled with foamy macrophages, best seen in a CD 68 stain. Verruciform xanthoma was diagnosed. Knowledge of this benign diagnosis may prevent an overly hasty, aggressive approach, since the differential diagnosis of penile carcinoma requires much more radical therapy, and mutilating penile surgery is associated with considerable psychosexual distress for patients.

## Anamnese

Ein 59-jähriger männlicher Patient stellte sich in unserer Klinik mit einem seit ungefähr 1 Jahr wachsenden Tumor an der Glans penis vor. Kurz vor dem Entstehen des Tumors habe er an dieser Stelle eine kleine Verletzung mit leichter Blutung gehabt. Es bestanden weder Schmerzen noch Juckreiz. Bei dem Patienten waren keine Vorerkrankungen bekannt, und er nahm keine Dauermedikation ein. Zuletzt habe er vor 3 Jahren ungeschützten Geschlechtsverkehr gehabt. Extern bereits unauffällig waren Treponema-pallidum-IgG- und IgM-Titer, sowie der Venereal Disease Research Laboratory-Test, HIV-Test und Hepatitis-C-Serologie.

## Klinischer Befund

**Figure 1:**
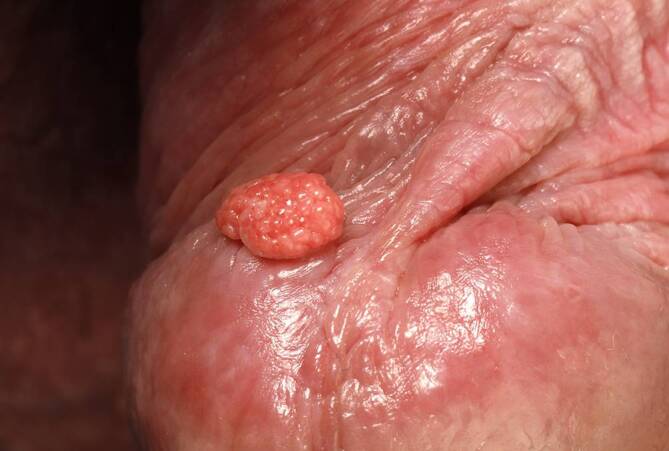

Bei der körperlichen Untersuchung zeigte sich ein gelblich-rötlicher, verruköser Tumor an der rechten Seite des Frenulums an der Glans penis. Dieser hatte eine glatt-spiegelnde Oberfläche ohne Ulzeration (Abb. 1).

## Therapie und Verlauf

Zum sicheren Ausschluss eines malignen Tumors erfolgte eine Flachexzision des Knotens in Lokalanästhesie. Es wurde eine sekundäre Wundheilung angestrebt. Der Patient stellte sich jedoch zu keiner Verlaufskontrolle mehr vor.

## Histologie

**Figure 2:**
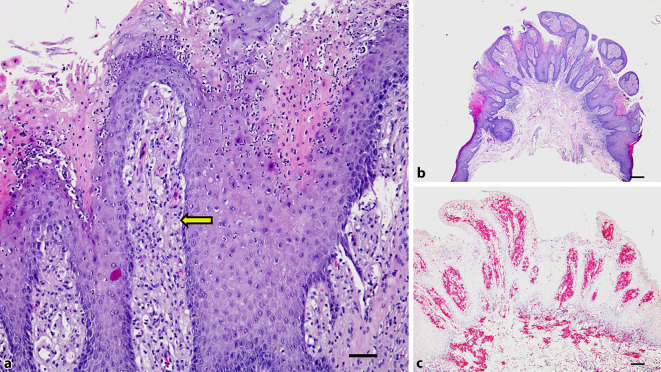

Bei der histologischen Aufarbeitung zeigte sich ein umschriebener, symmetrischer polypöser und verruköser Tumor (Abb. 2b). Die Epidermis war psoriasiform akanthotisch verbreitert mit Auflagerung einer teils kräftig eosinophilen, parakeratotischen Hornschicht. In dieser waren neutrophile Granulozyten eingeschlossen (Abb. 2a). Im Bereich des Epithels fanden sich keine Kern- und Zellatypien und auch keine suprabasalen Mitosen. Die Papillarkörper waren prall angefüllt mit zahlreichen Makrophagen (Abb. 2b, *Pfeil*). Begleitend fanden sich mäßig dichte lymphohistiozytäre Infiltrate. Eine CD68-Immunfärbung markiert die Makrophagen mit schaumigem Zytoplasma (Abb. 2c). Eine periodic-acid-Schiff(PAS)-Reaktion verblieb ohne Pilznachweis. Typisch für das verruciforme Xanthom sind die mit Schaumzellen prall gefüllten Papillarkörper sowie die beeindruckend psoriasiform konfigurierten Reteleisten der Epidermis mit neutrophilen Granulozyten im rötlich erscheinenden Stratum corneum.

## Diagnose und Diskussion

Wir diagnostizierten ein verruciformes Xanthom und somit einen gutartigen Tumor.

Das verruciforme Xanthom wurde erst 1971 von W.G. Shafer erstbeschrieben. Bei den Patienten liegen trotz des irreführenden Namens keine Fettstoffwechselstörungen vor. Es ist ein meist solitärer, benigner, normalerweise schmerzloser Tumor, der bevorzugt an der Mundschleimhaut zu finden ist [1]. Es finden sich jedoch auch Berichte über das Auftreten von verruciformen Xanthomen am Penis und an der Vulva [2, 5].

Wie in diesem Fall ebenfalls anamnestisch belegt, wird davon ausgegangen, dass der Entstehung meist ein degenerativer oder traumatischer Epithelschaden vorangeht [4]. Obwohl teilweise beschrieben, scheint eine Assoziation mit HPV-Infektionen nicht die Regel zu sein [9]. Die genaue Ätiopathogenese bleibt weiterhin unklar. Eine Exzision ist in der Regel jedoch eine kurative Therapie [8].

Verruciforme Xanthome können sich flach, papillomatös oder auch verrukös darstellen und werden klinisch häufig als Condyloma acuminatum eingeschätzt. Histologisch zeichnen sie sich durch eine Akanthose mit ausgeprägt verlängerten Reteleisten und mit Schaumzellen angefüllten Papillen aus [7].

Die ausgeprägte psoriasiforme Akanthose mit parakeratotischer Verhornung und Durchwanderung durch neutrophile Granulozyten stellt eine Karikatur des Bildes einer Psoriasis dar und kann in der Differenzialdiagnose verwirren. Das Bild des flammenzungenartig aufsitzenden eosinophilen Keratins des Stratum corneum auf einem verrukösen Tumor wird zudem gelegentlich als „wart on fire“ beschrieben [10, 11]**.**

Unilaterale Läsionen insbesondere in den Hautfalten und im Genitalbereich (Ptychotropismus) mit histologischem Bild des verruciformen Xanthoms sind als CHILD-Nävus charakteristisches Teilsymptom des CHILD(congenital gemidysplasia with ichthyosiform nevus and limb defects)-Syndroms bei Mädchen. Dieses zeichnet sich weiterhin durch variabel ausgeprägte ipsilaterale Extremitäten‑, Organdefekte und Defekte des zentralen Nervensystems aus. Bei geringer klinischer Ausprägung können die Hautveränderungen jedoch beispielsweise mit einer Psoriasis verwechselt werden. Der Vererbung erfolgt x‑chromosomal dominant. Männliche Träger des Gendefekt versterben schon intrauterin [6].

Eine wichtige Differenzialdiagnose von wachsenden Tumoren an der Glans penis sind zudem Läsionen aus dem Spektrum der Plattenepithelkarzinome. Das Peniskarzinom ist ein – insgesamt seltenes – Plattenepithelkarzinom, das meist im Bereich der Glans penis entsteht. Karzinome der Schafthaut sind extrem selten. Ungefähr 50 % der Peniskarzinome sind HPV-assoziiert [8]. Bis vor einigen Jahren wurden Peniskarzinome noch radikal mit mindestens 2 cm Sicherheitsabstand operativ entfernt, was zumeist einer Teilamputation des Penis gleichkam [3].

Sie können sich unter anderem ebenfalls papillomatös oder auch verrukös als Riesenkondylome darstellen. Histologisch zeigen sie jedoch ein in die Tiefe infiltrierendes Wachstum, unterschiedliche Grade von Entdifferenzierung, Zell- und Kernatypien sowie Mitosefiguren [12].

## Fazit für die Praxis

- Beim verruciformen Xanthom handelt es sich um einen benignen Tumor, der vornehmlich an der oralen und genitalen Schleimhaut auftritt.
- Er ist mit einer Exzision bereits ausreichend therapiert.
- Eine Untersuchung der Blutfette ist nicht vonnöten.
- Unilaterale Läsionen mit gleichartigem histologischem Bild sind als CHILD-Nävus charakteristisch für das CHILD-Syndrom bei Mädchen.
- Plattenepithelkarzinome der Genitalschleimhaut können sich initial klinisch ähnlich darstellen.
- Die Kenntnis dieser gutartigen Differenzialdiagnose sollte zu bedachtem Vorgehen anregen, um allzu mutilierende Penisoperationen und die damit einhergehenden psychosexuellen Belastungen zu vermeiden.
